# Cellulite des organes génitaux externes chez un homme au Mali

**DOI:** 10.48327/mtsi.v3i4.2023.428

**Published:** 2023-10-02

**Authors:** Moussa KEITA, Sanou Khô COULIBALY, Abdoulaye DIARRA, Sanra Déborah SANOGO

**Affiliations:** 1 Clinique privée « Néné », Kati, Mali; 2 Faculté de médecine et dodontostomatologie, Université des sciences, des techniques et des technologies de Bamako (USTTB), Mali; 3 CHU de Kati, Mali; 4 CHU du Point G, Bamako, Mali

**Keywords:** Cellulite, Pénis, Scrotum, Gangrène de Fournier, Diabète, Mali, Afrique subsaharienne, Cellulite, Penis, Scrotum, Fournier's gangrene, Diabetes Mali, Sub-Saharan Africa

## Abstract

L'observation présentée est celle d'une cellulite des organes génitaux externes (gangrène de Fournier) chez un diabétique âgé de sexe masculin hospitalisé à Kati au Mali. La rapidité d’évolution et la gravité de ce syndrome sont détaillées.

## Abstract (en)

The observation presented is that of cellulitis of the external genitalia (Fournier's gangrene) in an elderly diabetic male hospitalized in Kati in Mali. The speed of progression and severity of this syndrome are detailed.

Un homme de 74 ans ayant pour principal antécédent médical un diabète non insulino-dépendant connu depuis 8 ans a été hospitalisé dans une clinique de Kati pour une tuméfaction douloureuse du scrotum d'odeur fétide et un œdème de la verge évoluant depuis 3 semaines. À l'admission, le patient était agité et présentait une tuméfaction infiltrée, ulcérée, nécrosante du scrotum avec une crépitation, un suintement purulent, fétide du scrotum et un œdème de la verge (Fig. 1, 2 et 3). L’état général était altéré avec une pâleur cutanéo-muqueuse et palmo-plantaire. On observait également un globe vésical ainsi qu'une fièvre à 40 °C. L'indice de masse corporelle était de 21,45 kg/m^2^. L'anamnèse précisait que le début de la symptomatologie avait été marqué par des douleurs testiculaires bilatérales intenses et répétitives associées à des brûlures mictionnelles et à une dysurie évoluant en rétention aiguë, ainsi que par l'apparition d'une odeur fétide au niveau du scrotum. Le diabète de type 2 était non suivi et non traité par des antidiabétiques classiques, mais le patient prenait depuis plusieurs années pour ce diabète un traitement traditionnel par des plantes médicinales non identifiées. Aucune prise de corticoïdes n’était notée. Le bilan biologique montrait un taux d'hémoglobine à 9 g/dL, un taux d'hématocrite à 27%, une hyperleucocytose supérieure à 21 G/L, une thrombopénie à 9 G/L, une hyperglycémie à 3 g/L, une créatinine à 380 pmol/L et une élévation de la CRP. Les sérologies VHB et VIH étaient négatives. Un examen cytobactériologique des urines avec un antibiogramme mettait en évidence une infection urinaire à *Escherichia coli* sensible à l'amoxicilline + acide clavulanique. Faute de moyens financiers de la famille du patient, les hémocultures (62 euros) et l’échographie (76 euros) n'ont pas été pratiquées. Le diagnostic d'une cellulite des organes génitaux externes correspondant au syndrome de gangrène de Fournier était porté cliniquement devant un syndrome infectieux sévère associé à un aspect infiltré du tissu cellulaire sous-cutané scrotal avec crépitation et écoulement fétide favorisé par un diabète mal équilibré. Les diagnostics différentiels de traumatisme du scrotum, d'hydrocèle, d'anasarque ayant entraîné un œdème du scrotum étaient écartés par l'examen clinique. Malgré une prise en charge précoce par une antibiothérapie probabiliste à large spectre (amoxicilline + acide clavulanique, 2 g par jour, en intraveineux), la pose d'une sonde vésicale évacuatrice, la réhydratation hydroélectrique, l'injection d'insuline d'action rapide, la transfusion de sang total et le transfert dans un centre hospitalier, le patient décédait en 24 heures du fait d'un choc septique.

**Figure 1:**
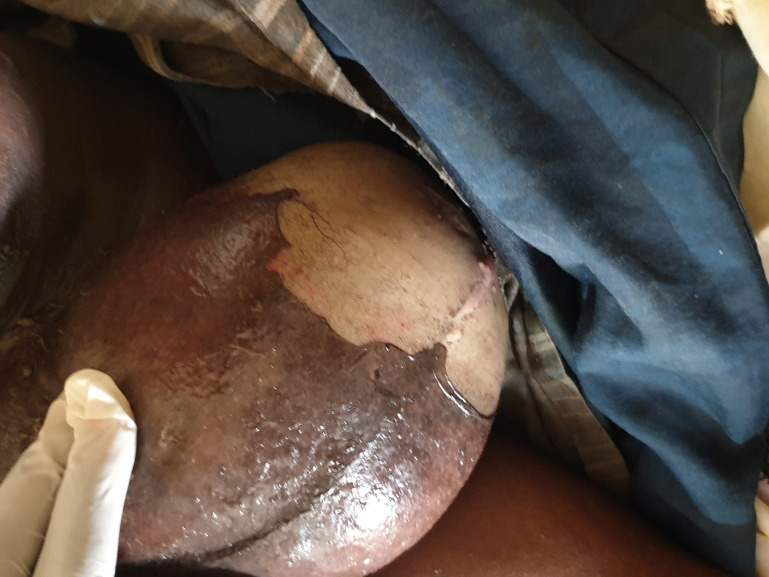
Tuméfaction, décollements cutanés et lésions purulentes du scrotum Swelling, skin separation and purulent lesions of the scrotum

**Figure 2:**
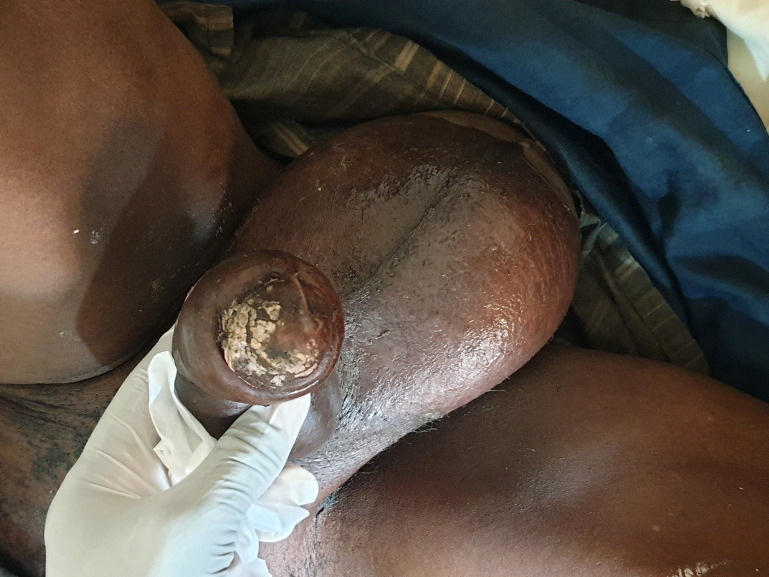
Œdème et ulcérations de la verge et du scrotum (1) Edema and ulcerations of the penis and scrotum (1)

**Figure 3:**
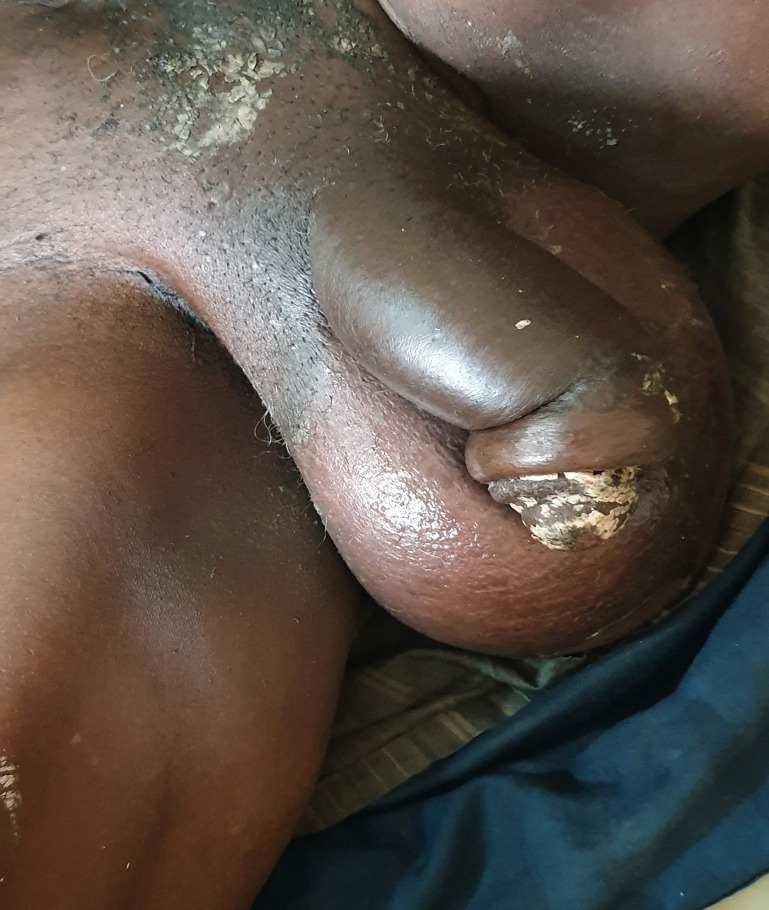
Œdème et ulcérations de la verge et du scrotum (2) Edema and ulcerations of the penis and scrotum (2)

Au Mali comme dans d'autres pays à ressources limitées, les cellulites infectieuses sont une complication fréquente du diabète. Les facteurs favorisants sont la méconnaissance du diabète, l'absence de son traitement ou son mauvais équilibre et le retard à la prise en charge des portes d'entrée cutanées. Ces cellulites touchent surtout les membres inférieurs, en particulier les pieds, ou la région cervico-faciale. La cellulite des organes génitaux externes est plus rare, mais des cas ont été rapportés au Mali et dans d'autres pays d'Afrique [1, 3]. Qu'il s'agisse de cellulite, de fasciite ou de gangrène, le terme de gangrène de Fournier est habituellement utilisé pour ces infections « foudroyantes » des organes génitaux externes [1]. Outre sa localisation, les particularités de ce syndrome sont une porte d'entrée bactérienne cutanée, urinaire ou digestive locorégionale, le polymicrobisme (entérobactéries, streptocoques, staphylocoques, anaérobies…) et surtout l’évolution très rapide des séquences « infection cutanée-cellulite-fasciite-gangrène » engageant en quelques heures le pronostic vital du fait du choc septique et en cas de diabète d'une acido-cétose, comme constaté dans notre observation. En dehors du diabète, les facteurs favorisant la gangrène de Fournier sont le grand âge et l'obésité comme dans notre observation, l'immunodépression et l'infection par le VIH [4]. Les hommes sont 10 fois plus atteints que les femmes. Le traitement médico-chirurgical est urgent, associant un débridement et une antibiothérapie à large spectre couvrant les bactéries anaérobies. La mortalité est élevée, de l'ordre de 20 à 80% selon la rapidité de la prise en charge médico-chirurgicale et la nature des comorbidités.

## Contribution des auteurs

Moussa KEITA: conception de l’étude, rédaction, co-rédaction et validation du protocole, analyse des données, interprétation des résultats.

Sanou Khô COULIBALY: conception de l’étude, relecture et validation du manuscrit

Abdoulaye DIARRA: conception de l’étude, rédaction, co-rédaction et validation du protocole, analyse des données, interprétation des résultats.

Sanra Déborah SANOGO: relecture et validation du manuscrit.

## Liens d'intérêts

Les auteurs ne déclarent aucun conflit d'intérêts.
